# The Relations of Materia Medica and Therapeutics to Stomatology

**Published:** 1896-05

**Authors:** Russell H. Cool

**Affiliations:** Oakland, Cal.


ARTICLE IX.

### THE RELATIONS OF MATERIA MEDICA AND THERAPEUTICS TO STOMATOLOGY.

BY RUSSELL H. COOL, D. D. S., OAKLAND, CAL.

Read before the California State Dental Association, July, 1895.

To me the most uncomplimentary definition of the word "dentist" is: "A man who takes care of and treats people's teeth;" yet there are many practicing our profession to whom that narrow definition would properly apply, and, in fact, it applies more than it should to the majority of stomatological practitioners. There is a tendency, in studying a specialty, for the student, in striving after the mastery of details, to restrict his horizon, to narrow his activity, to lose sight of the fact that the subject of his special study is one of the component parts of a great and complex whole, and that, if he hopes for the mastery of the *part*, he must know something of that cosmos of which it is one of the elements.

In dentistry the wonderful advancement of mechanical inventions has been, strange to say, one of the factors in producing this narrowness of which I speak. We are apt to devote our entire activity and effort to an endeavor to become great operators, skilled prosthetic workmen, artists in orthodontia; and we are apt to forget that the teeth are but part of that great, wonderful, throbbing engine—that complex system of moral, mental and physical force—which we call man. Let me not be considered as decrying the purely mechanical branches of our profession. By all means let our lives be devoted to necessary efforts to master them; but let us go farther and higher also. Let us remember that we have nervous conditions to deal with sometimes, as well as the eradication of decalcified tissue; that sometimes there is a sensitive, quailing invalid behind the cavity that is being artistically filled. We should have in mind our duty to the patient, and know that often we can relieve him more by systemic treatment than by any local manipulation, however skillful it may be, upon the teeth themselves.

I shall endeavor to give you the result of my experience in practice regarding the use of certain drugs in nervous conditions, in cases of excessive hemorrhage, anesthesia and the treatment of collapse and shock.

The study of the human face becomes absolutely unavoidable in successful practice. One patient that an injury will produce no visible effect upon will be followed by another upon whom a much slighter operation will produce a serious and enduring impression, and yet both of these patients are apparently in perfect health; still there is an unlikeness in their temperament, which should be our guide in calculating the effect of an operation.

In handling extremely nervous patients I believe it our duty to use any and all remedies at our disposal in an intelligent manner. Aromatic spirits of ammonia, administered previous to a tedious sitting, will often have a pleasant effect; also antikamnia, or a combination of antikamnia and codein. Phenacetin, or phenacetin and caffeine, acetanilide, and antipyrin, or, in some cases, morphina sulphate, or, in conjunction with the sulphate, atropine, one-fourth grain of the former and one-hundred-and-fiftieth grain of the latter, can be used advantageously, and a patient that would be a suffering, howling torment to the operator will become as docile and quiet as a lamb. Extreme cases come to us, and we should be as equal to an emergency as any surgeon. No surgical operation upon the human body is free from danger.

The first and most important of the consequences of any operation is hemorrhage. Though with every surgical operation there must always be a small amount of blood lost, it is the aim of the operator to lose as little as possible. The more the hemorrhage the slower the repair; and the less blood is lost the quicker the repair; also, in proportion to the loss of blood, shock takes place.

In continuance of this paper, I wish to draw attention to those remedies most important in *hemorrhage*, and anesthesia, and shock—those that are most energetic in producing a decided effect without loss of time. Here is a patient who has lost a certain amount of blood; is in a condition of such questionable nature that the operator is uncertain whether the subject will live or die, and sometimes these patients die. Why? Because the proper remedies are not administered. This frequently is the result of the ignorance of those who are in attendance. I have listened to a number of articles upon hemophila and have been surprised that the paper, or the discussion which followed, did not touch upon the systemic treatment of these cases. With the experience I have had, with the study I have given to general medicine, with what practical information I have gleaned from association with the medical profession, I have concluded that many deaths could have been prevented by the proper administration of medicines.

There is probably no one present who has not had experience with anesthetics when you have been compelled to perform a surgical operation within the mouth or upon the associated part. How many of you have found your patient with rapid respiration, rapid or intermittent pulse, patient filled with fear of the consequences of your efforts to repair some pathological condition. Such patients are most frequently anesthetized without preliminary preparation, and are frequently the source of great anxiety to the operator and the consulting physician. I have myself taken part in cases where the lips have turned pale, even purple, the finger nails turned black, a cold perspiration sprang out over the entire cutaneous surface, respiration ceased, pulse *nil.* and the patient apparently in a moribund condition, where to the casual observer death must certainly take place; and yet, with the hypodermic use of whisky, aromatic spirits of ammonia, digitalis, nitro-glycerin and artificial respiration, the circulation and respiration of this patitient speedily took place and the patient returned to life. These and kindred agents I wish to dwell upon, and thus occupy a few moments of your valuable time.

*Alcohol.*—May be injected at a temperature of 90 degrees, 30 drops to 1 dram, without sloughing. A temperature below this or above may produce sloughs. Flood made two hundred injections of whisky in coffee in four hours with complete success, though at the same time he injected 10-to-20 minim doses of tincture of belladonna, which may have contributed to the result.

*Amyl-Nitris.*—Sedative and anti-spasmodic; lower temperature by ozonizing the blood; relaxes arterial system; reduces arterial pressure. 16 to 80 minims have been injected in a dog, and 90 minims in a man, but I consider 10 minims an unsafe dose hypodermically.

In a formula of 10 parts amyl-nitrite, alcohol 30 parts glycerine 60 parts; total 100 parts, 8 to 15 minims should be injected every hour, as the condition of the patient, should warrant—that is, the absence of color to the face, a small and frequent wiry pulse.

*Nitro-Glycerinum.*—A powerful poison. 1 to 6 drops of 1 per cent solution produces fulness, quickness of pulse, diminution of arterial pressure; similar in its effect to amyl-nitrite, and can be used in hemorrhage, in stimulating heart action, without causing perceptible increase of arterial pressure; hence a remedy of much importance in hemorrhage, because it increases the vital forces without producing arterial fullness, which would result in further hemorrhage and possibly death. Would advise the injection of 1 drop of 1 per cent solution a few minutes before the injection of cocaine. Would suggest also its use to the extent of 1, 2 or three drops in all cases of collapse after the administration of an anesthetic, or any surgical operation, but would recommend in addition to this remedy the speedy hypodermic injection of aromatic spirits of ammonia, whisky and digitalis. The three latter agents may be used hypodermically in from ½ to 1 drachm doses and these doses may be repeated every half-hour, if the patient is in extreme collapse, but if in ordinary cases of hemorrhage, with but slight shock to the patient, 15 minims each of these three remedies at a temperature of 90 degrees, with injection of 2 or 3 minims of nitroglycerine and inhalations of nitrite of amyl, are sufficient to stimulate the vital forces and bring the vital organism back to its normal condition.

The question arises in our minds: What parts of the human anatomy should we avoid in the use of the hypodermic needle, and of what parts should we make use? the places to be avoided in puncturing are veins, inflamed parts and bony prominences, to be more expressive, it would not be wise, if you desire to give any medicated injections, to insert the needle through the skin on the inside of the tibia, on the dorsal surface of the foot, on the back of the hand or fingers, on the scalp, on the forehead, over the olecranon process or posterior to the ear. These parts are principally bony tissues, covered mostly by skin, and almost devoid of lymphatics, the latter being eminently necessary in taking up a medication and carrying it to the central organs to procure the required effect.

Having considered the points on human anatomy to be avoided, I will speak now of those regions which, in my mind, are the most appropriate for the assimilation of agents that we desire to equalize the circulation and to stimulate the nervous system: The external part of the arm, away from the principal blood-vessels; the outer side of the thigh, away from the principal blood-vessels; in the abdomen, where there are practically no blood-vessels, and in the chest tissues, where there is also an absence of important vessels.

Now; a word about the syringe, which should be thoroughly sterilized. After drawing the required amount of liquid into the syringe, shall we immediately insert the needle through the skin and inject the fluid therein? Most certainly not, because in drawing a fluid from a receptacle a certain amount of air must certainly be in the barrel of the syringe; hence, it will be necessary to place the syringe in a perpendicular position, the needle uppermost, and the piston must then be pushed forward until all the air is forced from within, and one drop of the fluid appears. I believe that lack of attention to this particular point is the cause of a great deal of sloughing and disagreeable effects, and the injection of a minute portion of air has resulted fatally.

Also one word regarding the preliminary treatment of anesthetic cases, or those particular cases where, with the usual observations, we frequently find the pulse abnormally rapid, the patient timid, the respirations rapid, and all indications present of a slight shock before any surgical interference. I have concluded that there are medicines in the pharmacopeia that will destroy those depressive conditions and permit us to continue to operate in safety—considering that the heart and kidneys are in a normal condition. I am in the habit of administering ¼ grain of morphine by the mouth one hour before an operation. 15 or 20 grains of bromide of potash have also a happy result in quieting the nervous system, and when administered with brandy we have the cerebral sedative and cardiac stimulant. Chloral 5 grains, morphine ¼ grain, brandy 1 ounce, have with me destroyed, in the majority of cases, the usual nervous symptoms that are unpleasant to me as a precedent to any operation. These same agents may be administered with beneft to our patients when operations are performed without anesthesia.

I do not think it necessary to apologize for not dwelling on and explicitly explaining the doses of digitaline ergotine, cocaine, atropia, strychnia and various other important agents useful in conjunction with these extreme cases. The dispensatory and pharmacopeia will explain their uses and doses.

In conclusion, I do not wish the members of this association to think that it is my advice for the dentist to usurp the office of the physician, but I do maintain that a thorough knowledge of the matter under deliberation is essential to our specialty, and may, at the most unforseen time, be the means of saving a life.—*Western Dental Journal.*

